# Elevated fasting glucose level increases the risk of fatty liver disease: a 10-year study of 31,154 individuals

**DOI:** 10.1186/s12876-022-02615-0

**Published:** 2022-12-16

**Authors:** Jiang Deng, Zhiyi Han, Hailing Tang, Cong Yao, Xiaoling Li, Jingyuan Xu, Mimi Zhou, Xin Xing, Fangxiong Wu, Jianning Li, Xiaolan Lu, Haitao Shi

**Affiliations:** 1 Department of Gastroenterology, The Second Affiliated Hospital of Xi’an Jiaotong University, Xi’an, 710004 China; 2 Nursing Department, The Second Affiliated Hospital of Xi’an Jiaotong University, Xi’an, 710004 China; 3 Karamay Center Hospital of Xinjiang, Karamay, 834099 China; 4 Division of Gastroenterology, Xi’an Central Hospital, Xi’an, 710004 China; 5 Department of Gastroenterology, First Affiliated Hospital of Xi’an Medical University, Xi’an, 710077 China; 6 Department of Gastroenterology, Shanghai Pudong Hospital of Fudan University, Shanghai, 201399 China

**Keywords:** Fatty liver disease, Fasting blood glucose, Triglycerides, Body mass index, Dysglycemia

## Abstract

**Objectives:**

Dysglycemia promotes the occurrence of fatty liver disease (FLD). However, the process is unclear. This study aimed to analyze the median time-to-onset, cumulative prevalence and influencing factors for the occurrence of FLD in people undergoing routine screening and evaluation.

**Methods:**

Data from Karamay Central Hospital (September 2008–April 2017) were analyzed. Survival analysis was performed to calculate the median time and cumulative prevalence of FLD associated with normal and elevated fasting blood glucose (FBG) levels. Cox proportional hazards model was used to determine risk factors.

**Results:**

A total of 31,154 participants were included in the two cohorts of this study, including 15,763 men. The mean age was 41.1 ± 12.2 years. There were 2230 patients (1725 male) in the elevated FBG group, the median age was 53 years (range 21–85 years), the median time-to-onset of FLD was 5.2 years. The incidence of FLD was 121/1000 person-years, and the 1-, 3-, 5-, and 7-year prevalence rates were 4%, 30%, 49%, and 64%, respectively. The normal FBG group included 28,924 participants (14,038 male), the median age was 40 years (range 17–87 years), and the corresponding values were as follows: 8.3 years, 66/1000 person-years, and 3%, 16%, 28%, and 41%, respectively. The Cox proportional hazards analysis revealed that age, blood pressure, FBG, body mass index and triglycerides were independent influencing factors for FLD in individuals (*P* < 0.05).

**Conclusions:**

Elevated FBG levels increase the risk of FLD and should be treated promptly.

**Supplementary Information:**

The online version contains supplementary material available at 10.1186/s12876-022-02615-0.

## Introduction

Dysglycemia increases the risk of fatty liver disease (FLD). Approximately 70% of patients with type 2 diabetes mellitus (T2DM) have non-alcoholic fatty liver disease (NAFLD), and the risk of nonalcoholic steatohepatitis (NASH) is higher in people with diabetes than in the general population [1, 2]. A previous meta-analysis involving 49,419 people with T2DM (52.9% men) reported a global NAFLD prevalence of 55.5% (95% confidence interval [CI] 47.3%–63.7%) among patients with T2DM. Moreover, in 10 studies that reported the prevalence of NASH, the global rate among people with T2DM was 37.3% (95% CI 24.7%–50.0%) [3].

According to a 2013 nationally representative cross-sectional survey of 170,287 people in China, the rate of pre-diabetes in adults was 35.7% (95% CI 34.1%–37.4%) [4]. People with pre-diabetes have a higher risk of developing diabetes, FLD [5, 6]. However, to our knowledge, there are no studies on the time to FLD incidence in patients with elevated fasting glucose (FBG) levels [7–9]. Therefore, the data of 31,154 people undergoing routine screening and evaluation in Karamay Center Hospital in last 10 years were analyzed retrospectively, to analyze the median time, cumulative prevalence, and influencing factors on the occurrence of FLD, to provide reference for the prevention and treatment of related diseases.

## Methods

### Patients and study design

We retrospectively evaluated 128,542 individuals (76,001 men and 52,541 women) who underwent routine screening and evaluation between September 2008 and April 2017 at Karamay Central Hospital, which is a large public hospital in Karamay City catering to workers, teachers, and other professionals. FLD was diagnosed by ultrasound, the typical manifestations of FLD are enlargement of liver and blunt edge Angle. The near field echo of liver was diffusely enhanced, higher than that of spleen and kidney, while the far field echo was weakened. Hepatic duct structure is not clear. Some physicals are examined once a year, some twice a year.

The inclusion criteria were as follows: in the elevated FBG group (n = 2,230), participants who had elevated FBG levels (> 6.2 mmol/L) but no FLD at baseline. In the normal FBG group (n = 28,924), participants who had normal FBG levels (≤ 6.2 mmol/L) and no FLD at baseline. The threshold of FBG level was determined by the reference values used by the hospital.

The exclusion criteria were as follows: (1) having data of fewer than two routine screening and evaluations, (2) presence of liver cirrhosis, liver tumor, liver hydatid disease, liver transplantation, polycystic liver, or any other liver diseases that may influence baseline FBG levels. Patients were considered lost to follow-up when any of these diseases occurred during the study period.

### Outcomes and risk factors of interest

All data per participant were considered. The time and frequency of routine screening and evaluation varied for each participant; the included participants were observed dynamically and without an established or consistent pattern. Survival analysis was performed to determine the median time-to-onset of FLD and the 1-, 3-, 5-, and 7-year prevalence rates of FLD in the normal FBG group and the elevated FBG group. We compared annual survival rates using the Z-test to identify the risk of FLD in the two groups. Because the risk is different for men and women at different ages, male and female were stratified and cox regression analysis was performed, age, blood pressure, body mass index (BMI), FBG, and triglycerides (TG) were included.

This is a limited evaluation from 2008 to 2017. Some people have a lot of examination items, while others have only the most basic examination items such as ultrasound, BMI, BP, FBG and TG. Therefore, BMI, BP, FBG and TG and other factors that we considered to be the most relevant were selected for analysis.

### Index calculation and reference values

Unlike the international and Asian standard values, the standard values for overweight and obesity in China are 24 kg/m^2^ and 28 kg/m^2^; hence, high BMI (kg/m^2^) was defined as BMI ≥ 24 kg/m^2^ in this study. Systolic blood pressure > 140 mmHg and diastolic blood pressure > 90 mmHg were considered elevated.

FBG > 6.2 mmol/L and TG > 1.83 mmol/L were considered elevated. These were determined using the range of reference values provided by the local hospital.

### Statistical analysis

All statistical analyses were performed using IBM SPSS Statistics version 23.0 for Windows (SPSS Inc., Armonk, NY, USA). Categorical variables are reported as counts and ratios. Rates were compared, and trends were tested using the χ^2^ test. The disease course was examined using survival analysis. Life tables were used to calculate the median time-to-onset of FLD and the 1-, 3-, 5-, and 7-year prevalence rates of FLD in patients with elevated and normal FBG levels. Cox proportional hazards model was used to determine risk factors. The Z-test was used to compare annual survival rates. The Breslow and log-rank tests were used to compare short- and long-term differences, respectively, in survival curves between the two groups. *P*-values < 0.05 were considered statistically significant.

## Results

### Comparison of baseline data

A total of 31,154 people were included in the two cohorts of this study, including 15,763 men. The mean age was 41.1 ± 12.2 years. The number of male participants, those aged ≥ 50 years, and participants with elevated BMI, blood pressure, and TG levels were significantly higher in the elevated FBG group than in the normal FBG group (*P* < 0.001; Table 1) (Additional file 1).

**Table 1:** Comparison of elevated and normal FBG group

Characteristics	Total	Elevated FBG group	Normal FBG group	*P*
Total	31,154	2230	28,924	
*Gender*				< 0.001
Male	15,763 (50.6%)	1725 (77.4%)	14,038 (48.5%)	
Female	15,391 (49.4%)	505 (22.6%)	14,886 (51.5%)	
*Age, years*				< 0.001
< 50	24,818 (79.7%)	896 (40.2%)	23,922 (82.7%)	
≥ 50	6336 (20.3%)	1334 (59.8%)	5002 (17.3%)	
*BMI, kg/m*^*2*^				< 0.001
Not high	18,618 (59.8%)	806 (36.1%)	17,812 (61.6%)	
High	12,536 (40.2%)	1424 (63.9%)	11,112 (38.4%)	
Mean ± standard deviation		25.0 ± 2.9 kg/m^2^	23.2 ± 3.0 kg/m^2^	
*Blood pressure*				< 0.001
Not high	24,859 (79.8%)	1116 (50.0%)	23,743 (82.1%)	
High	6295 (20.2%)	1114 (50.0%)	5181 (17.9%)	
*Triglycerides*				< 0.001
Not high	26,226 (84.2%)	1508 (67.6%)	24,718 (85.5%)	
High	4928 (15.8%)	722 (32.4%)	4206 (14.5%)	

FBG > 6.2 mmol/L, BMI ≥ 24 kg/m^2^, systolic blood pressure > 140 mmHg and diastolic blood pressure > 90 mmHg, triglycerides > 1.83 mmol/L were considered elevated. Data are presented as n (%)

*BMI* body mass index, *FBG* fasting blood glucose

### Observation of the occurrence of FLD in individuals with normal and elevated FBG level

The median age of participants in the elevated FBG group was 53 years (range 21–85 years). The follow-up period ranged from 0.2 to 8.7 years, the median follow-up period was 2.7 years. The highest FBG level at the beginning and end of the study period was 29.0 mmol/L (median, 7.2 mmol/L) and 21.5 mmol/L (median, 7.3 mmol/L), respectively. The proportion of those lost to follow-up was 1.4% (31 cases of polycystic liver, liver transplantation, cirrhosis, splenomegaly, splenectomy, and liver tumor).

A total of 7211.0 person-years were recorded in the present study, during which 876 cases of FLD were observed, and 1323 participants remained free of FLD. The incidence density of FLD was 121/1000 person-years, while the cumulative incidence of FLD was 39.3%. Concurrently, the median time-to-onset of FLD was 5.2 years, and the 1-, 3-, 5-, and 7-years prevalence rates were 4%, 30%, 49%, and 64%, respectively.

The median age of the normal FBG group was 40 years (range 17–87 years). The follow-up period ranged from 0.1 to 8.7 years, the median follow-up period was 3.2 years, during which FBG levels for all participants remained within the normal range. The proportion of those lost to follow-up was 0.6% (179 cases of polycystic liver, liver transplantation, splenomegaly, cirrhosis, splenectomy, and liver tumor).

A total of 106,934.5 person-years were recorded, 7081 cases of FLD were observed, and 21,664 remained free of FLD. The incidence density of FLD was 66/1000 person-years, and the cumulative incidence of FLD was 24.5%. The median time-to-onset of FLD was 8.3 years, and the 1-, 3-, 5-, and 7-year prevalence rates were 3%, 16%, 28%, and 41%, respectively. In total, 14,464 people had normal TG levels, BMI, and blood pressure, the cumulative prevalence of FLD was 10.7% (1541 cases of FLD).

Among 7957 patients with FLD, ALT and AST were detected in 6680 of them, AST/ALT > 2 was found in only 263 patients (3.9%), AST > 40 U/L was found in only 18 patients. ALT/AST > 1 was found in 3836 patients (57.4%). Based on these preliminary calculations, the main form of FLD seen in the hospital was NAFLD, with ALD rarely diagnosed. NAFLD has now been renamed metabolic associated fatty liver disease, its diagnostic criteria are independent of the amount of alcohol consumed by a patient.

**Fig. 1:**
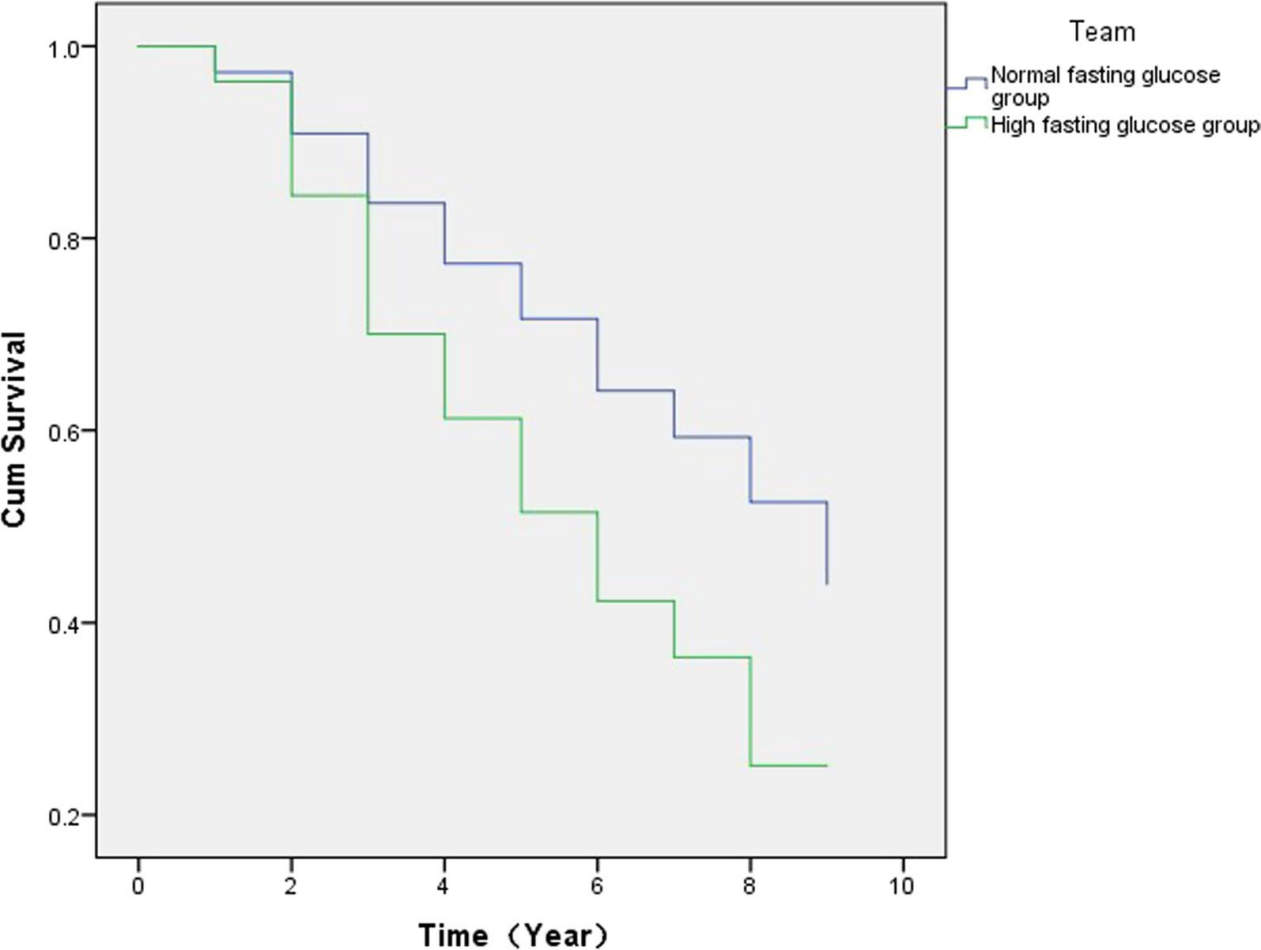
Survival analysis curve of fatty liver disease in people with elevated and normal fasting blood glucose

The Breslow and log-rank tests revealed that the short- and long-term risks of FLD were significantly higher in the elevated FBG group than in the normal FBG group (*P* < 0.001). The risk of FLD was already significantly higher in the first year following detection in the elevated FBG group than in the normal FBG group (*P* < 0.001; Fig. 1).

### The incidence of FLD in individuals with elevated FBG levels with different BMIs

BMI of normal and elevated FBG group were 23.2 ± 3.0 kg/m^2^ and 25.0 ± 2.9 kg/m^2^, respectively. The Breslow and log-rank tests revealed that, compared to normal BMI, low BMI in patients with elevated FBG did not affect the short-term risk of FLD; however, long-term risk was significantly reduced in this group (*P* = 0.029).

In the elevated FBG group, the short- and long-term risk of FLD in participants who were overweight or with obesity was significantly increased (*P* < 0.001) compared to participants with normal BMI. In emaciated patients with elevated fasting glucose, the prevalence of FLD was decreased over the years. However, there was no significant difference in the short-term risk (*P* = 0.076), and the long-term risk was significantly decreased (*P* = 0.029). Table 2.

**Table 2:** The incidence of FLD in individuals with elevated FBG with different BMIs

BMI, kg/m^2^	Male/female	Median onset period (years)	1-Year prevalence (%)	3-Year prevalence (%)	5-Year prevalence (%)	7-Year prevalence (%)
< 18.5	11/10	8.0	0	0	12	12
18.5–23.9	545/240	7.2	1	18	33	46
24–28	910/188	4.7	4	33	53	68
≥ 28	259/67	3.3	7	47	67	85

### The incidence of FLD in patients with elevated FBG levels in different age and sex groups

In the elevated FBG group, the cumulative prevalence of FLD was higher in men than in women (46.4% vs. 34.7%, *P* < 0.01) among those aged < 50 years. However, among the individuals aged ≥ 50 years, the cumulative prevalence of FLD was significantly higher in women than in men (45.5% vs. males 34.4%, *P* < 0.01; Table 3).

**Table 3:** The incidence of FLD in patients with elevated FBG in different age and sex groups

Characteristics	Total	Male	Female	*P*
< 50 years	43.3% (388/896)	46.4% (305/657)	34.7% (83/239)	< 0.01
≥ 50 years	36.6% (488/1334)	34.4% (367/1068)	45.5% (121/266)	< 0.01

### Cox regression model for the occurrence of FLD

Cox regression analysis was performed respectively because the risk of metabolic diseases was different in males and females at different ages.

The Cox proportional hazards analysis revealed that age, BP, FBG, BMI and TG level were independent influencing factors for the occurrence of FLD in individuals (*P* < 0.05). Elevated FBG level increases the risk of FLD. Tables 4 and 5.

**Table 4:** Cox regression model for the occurrence of FLD in male

Factors	*P*	HR	95% CI for HR
Age (years)	< 0.001	0.992	0.990, 0.994
BMI (kg/m^2^)	< 0.001	1.193	1.183, 1.204
BP (not high/high)	< 0.001	1.135	1.067, 1.206
TG (mmol/L)	< 0.001	1.161	1.144, 1.177
FBG (mmol/L)	0.006	1.028	1.008, 1.048

*BMI* body mass index, *BP* blood pressure, *TG* triglyceride, *HR* hazard ratio

**Table 5:** Cox regression model for the occurrence of FLD in female

Factors	*P*	HR	95% CI for HR
Age (years)	< 0.001	1.010	1.006, 1.014
BMI (kg/m^2^)	< 0.001	1.252	1.240, 1.265
BP (not high/high)	0.022	1.125	1.017, 1.244
TG (mmol/L)	< 0.001	1.374	1.332, 1.417
FBG (mmol/L)	< 0.001	1.063	1.032, 1.096

*BMI* body mass index, *BP* blood pressure, *TG* triglyceride, *HR* hazard ratio

## Discussion

The association between dysglycemia and FLD is well documented. Specifically, elevated FBG levels increase the risk of DM [10, 11] and FLD [5, 6, 12]. However, time and the influence of the number of risk factors on disease onset remains unclear [13]. This study is the first to show that the risk of FLD in the elevated FBG group was significantly higher than that in the normal FBG group during the first year following detection.

Dysglycemia increases the risk of FLD. Our research shows that, in the elevated FBG group, the median time-to-onset of FLD was 5.2 years. The incidence of FLD was 121/1000 person-years, and the 1-, 3-, 5-, and 7-year prevalence rates were 4%, 30%, 49%, and 64%, respectively. In normal FBG group, the corresponding values were as follows: 8.3 years, 66/1000 person-years, and 3%, 16%, 28%, and 41%, respectively. Dysglycemia is often concurrent with insulin resistance and decreased insulin sensitivity [14]; it can inhibit oxidative decomposition and utilization of free fatty acids in the liver and increase triacylglycerol levels. However, the liver has limited ability to metabolize lipids, and lipids accumulate in the liver cells to form FLD [15].

A prospective study of 2802 patients with T2DM followed for 2 years reported 813 (29.0%) patients with NAFLD [16]. The 3-year cumulative prevalence rate of FLD (30%) was similar to that in the elevated FBG group. The cumulative prevalence rate of FLD varied with different participants and follow-up period. Presently, the prevalence of dysglycemia is high, but disease awareness and treatment uptake rates are low. In the present study, due to the large sample size, we only measured FBG levels, other factors, such as postprandial glucose might also be associated with the risk of FLD. In real-world settings, individuals who undergo routine screening and evaluation and are not specifically diagnosed with diabetes will most likely to only undergo FBG detection tests.

Previous studies regarding dysglycemia and FLD were either cross-sectional studies or cohort studies with a short follow-up. Although they have reported on FLD incidence and cumulative incidence in this patient group, they did not consider the time-to-onset of FLD in this population [17–19]. The present study examined cumulative incidence over time and median onset of FLD in patients with elevated FBG, and the results support they need for managing FLD risks over time starting in the first year when elevated FBG levels are detected.

Age, BP, FBG, BMI and TG levels were independent factors for the occurrence of FLD in individuals. These findings are consistent with those of previous studies [20–23]. Metabolic syndrome is a group of complex metabolic disorders, including dysglycemia, increased BMI or waist circumference, essential hypertension, and other diseases. NAFLD is the manifestation of metabolic syndrome involving the liver [24]. Metabolic syndrome-related diseases have been shown to promote the development of FLD in a previous observational study involving 258 people with T2DM, where 167 (64.7%) participants had NAFLD; higher rate of obesity; and elevated liver enzymes, TG, and cholesterol were associated with NAFLD in patients with T2DM [25].

Although high BMI is associated with FLD, individuals who are emaciated can also develop FLD. When people reduce their protein consumption to lose weight, they tend to develop an apolipoprotein deficiency, which reduces the liver’s ability to remove TG and in turn promotes FLD. In most studies, somatotype is measured exclusively with the BMI, even though the waist-hip ratio and body fat distribution might be better indicators of visceral obesity than BMI; however, such data are rarely available. In the present study, the number of emaciated participants (n = 21) in the elevated FBG group was too small to allow for meaningful statistical analysis. Future studies should examine this association using larger samples. Non-obese FLD may also be associated with other factors such as high TG levels, high consumption of fructose, and high cholesterol diet intake.

The risk of FLD varies by age and sex, and may be related to lifestyle, social responsibilities and stress, and hormonal changes. Specifically, reduced estrogen level after menopause lead to an increased risk of FLD in women aged > 50 years. A previous cross-sectional study of rural-based women in Bangladesh reported that the prevalence of metabolic syndrome is 1.78 times higher in postmenopausal women than in premenopausal women; concurrently, the prevalence of hypertension and increased FBG and TG levels was significantly higher in postmenopausal women than in premenopausal women (*P* < 0.05) [26]. These findings suggest that age and sex are important risk factors for metabolic syndrome diseases, and thus, post-menopausal women should be monitored closely.

To the best of our knowledge, this is the first retrospective cohort study to analyze the median time-to-onset, cumulative prevalence, and risk factors for the occurrence of FLD in individuals. However, there are some limitations to this study. Ultrasonography is less sensitive in diagnosing FLD, but it is cost-effective, non-invasive, and is the most common in real-world settings. Other data such as disease history, alcohol intake, and postprandial blood glucose could not be supplemented, and the impact of the relevant data could not be further evaluated. A prospective multicenter study is required to provide more accurate estimates.

In general, our results suggest that elevated FBG increases the cumulative prevalence of FLD and shortens the duration of onset. Age, BP, FBG, BMI and TG were independent influencing factors for FLD in individuals. For both men and women, elevated FBG levels increase the risk of FLD and should be treated promptly.

## Supplementary Information

**Additional file 1.** The data from the study that elevated fasting glucose levels increase the risk of fatty liver disease.

## Data Availability

Data from the study has been uploaded as Additional file 1, but the availability of the data is limited and the data was used under the license of the current study and therefore cannot be made public. To obtain data, please contact the last corresponding author Haitao Shi.

## Abbreviations

FLD: Fatty liver disease
NAFLD: Non-alcoholic fatty liver disease
BMI: Body mass index
SBP: Systolic blood pressure
DBP: Diastolic blood pressure
ALT: Alanine aminotransferase
TBIL: Total bilirubin
FBG: Fasting blood glucose
TG: Triglycerides
TC: Total cholesterol
CI: Confidence interval
HR: Hazard ratio
